# Identification of Metabolism-Related Gene-Based Subgroup in Prostate Cancer

**DOI:** 10.3389/fonc.2022.909066

**Published:** 2022-06-16

**Authors:** Guopeng Yu, Bo Liang, Keneng Yin, Ming Zhan, Xin Gu, Jiangyi Wang, Shangqing Song, Yushan Liu, Qing Yang, Tianhai Ji, Bin Xu

**Affiliations:** ^1^ Department of Urology, Shanghai Ninth People’s Hospital, Shanghai Jiaotong University School of Medicine, Shanghai, China; ^2^ The Second Affiliated Hospital, School of Medicine, Zhejiang University, Hangzhou, China; ^3^ 174 Clinical College, Anhui Medical University, Hefei, China; ^4^ Department of Pathology, Shanghai Ninth People’s Hospital, Shanghai Jiaotong University School of Medicine, Shanghai, China

**Keywords:** prostate cancer, metabolism, immune, non-negative matrix factorization (NMF), The Cancer Genome Atlas (TCGA), International Cancer Genome Consortium (ICGC)

## Abstract

Prostate cancer is still the main male health problem in the world. The role of metabolism in the occurrence and development of prostate cancer is becoming more and more obvious, but it is not clear. Here we firstly identified a metabolism-related gene-based subgroup in prostate cancer. We used metabolism-related genes to divide prostate cancer patients from The Cancer Genome Atlas into different clinical benefit populations, which was verified in the International Cancer Genome Consortium. After that, we analyzed the metabolic and immunological mechanisms of clinical beneficiaries from the aspects of functional analysis of differentially expressed genes, gene set variation analysis, tumor purity, tumor microenvironment, copy number variations, single-nucleotide polymorphism, and tumor-specific neoantigens. We identified 56 significant genes for non-negative matrix factorization after survival-related univariate regression analysis and identified three subgroups. Patients in subgroup 2 had better overall survival, disease-free interval, progression-free interval, and disease-specific survival. Functional analysis indicated that differentially expressed genes in subgroup 2 were enriched in the xenobiotic metabolic process and regulation of cell development. Moreover, the metabolism and tumor purity of subgroup 2 were higher than those of subgroup 1 and subgroup 3, whereas the composition of immune cells of subgroup 2 was lower than that of subgroup 1 and subgroup 3. The expression of major immune genes, such as CCL2, CD274, CD276, CD4, CTLA4, CXCR4, IL1A, IL6, LAG3, TGFB1, TNFRSF4, TNFRSF9, and PDCD1LG2, in subgroup 2 was almost significantly lower than that in subgroup 1 and subgroup 3, which is consistent with the results of tumor purity analysis. Finally, we identified that subgroup 2 had lower copy number variations, single-nucleotide polymorphism, and neoantigen mutation. Our systematic study established a metabolism-related gene-based subgroup to predict outcomes of prostate cancer patients, which may contribute to individual prevention and treatment.

## Introduction

Prostate cancer (PCa) is the most common urological cancer among men in the United States (1). It is reported that in 2021, there were an estimated 250,000 new cases and 34,000 deaths (2). The 5-year prevalence of PCa is the highest globally, and its age-standardized mortality is the sixth highest (1). The clinical diagnosis of PCa mainly depends on digital rectal examination, serum prostate-specific antigen, and imaging examination (3). Usually, prostate biopsy for pathological examination is also required. Gleason score can help to evaluate the malignancy of PCa. Although radical prostatectomy has become the main strategy for resection of localized primary prostate tumors, more than one in five PCa patients inevitably progress to the advanced stage with a poor prognosis within 10 years (4, 5). Due to the high rate of bone metastasis, PCa patients shared an unfavorable prognosis (6). Other treatment methods, such as hormone therapy and chemoradiotherapy, are not satisfactory for the prognosis (7). In addition, PCa has high heterogeneity, which results in different prognoses of patients after treatment (8). Therefore, there is a need to deeply understand the potential mechanism leading to PCa progression and metastasis, and select specific subtypes to find the population who benefit the most.

The cell proliferation state in tumor progression involves the corresponding changes of cell metabolism (9), and metabolic reprogramming is considered to be a basic feature of cancer cells (9). Metabolism in tumors, such as the Warburg effect and glutamine metabolism, is significantly different from that in normal tissues (10, 11). There is increasing evidence that metabolic abnormalities are associated with poor prognosis of many tumor types (12). Fortunately, the screening of metabolic biomarkers can specifically detect abnormal changes in organisms to prevent malignant diseases with pathophysiological characteristics (13). The metabolic markers have been well displayed in a variety of tumors, such as hepatocellular carcinoma (14), colorectal cancer (15), endometrial cancer (16), and clear cell renal cell carcinoma (17), but research in PCa is still relatively scarce. Therefore, it is of great clinical significance to find a new metabolic marker to predict the prognosis of PCa.

In the present study, we systemically analyzed the profile of metabolism-related genes from The Cancer Genome Atlas (TCGA) and International Cancer Genome Consortium (ICGC). All patients could be grouped into three subgroups with different prognoses through non-negative matrix factorization (NMF) based on TCGA, which was validated in ICGC. Then we obtained differentially expressed genes (DEGs) and chronologically conducted functional analysis, gene set variation analysis (GSVA), and immune-related comprehensive analysis (**Figure S1**).

## Materials and Methods

### Acquisition and Processing of Raw Data

We downloaded the RNA-sequence data, clinical information, survival data, and somatic mutation data of TCGA prostate adenocarcinoma (PRAD) from UCSC Xena (https://xenabrowser.net/datapages/). According to clinical information corresponding to the sample, we only select these samples whose primary diagnosis is “adenocarcinoma, NOS” and sample type is “primary tumor” follow-up analysis. After the expression profile was reannotated, the expression data of all mRNAs were selected according to the human genome information contained in the HUGO Gene Nomenclature Committee (HGNC) database (18), and a new expression profile was obtained.

Then, we downloaded the gene expression files, sample information, and clinical information corresponding to PRAD-CA from the ICGC database, removed the normal samples (only PRAD samples were included), and sorted out the new expression profile data according to the HGNC database for subsequent verification and analysis.

### Screening of Metabolism-Related PRAD Genes

We cross-referenced maps of metabolic pathways with the Kyoto Encyclopedia of Genes and Genomes database to compile a comprehensive list of 2,752 genes encoding all known human metabolic enzymes and transporters (19). After intersecting with TCGA PRAD mRNA expression profile, we obtained the metabolism-related genes in PRAD. After that, we removed the genes with median absolute deviation (MAD) < = 0.5 to obtain metabolism-related PRAD genes.

### NMF and Survival Analysis

We performed survival-related univariate regression analysis on metabolism-related PRAD genes and selected the significant genes (P < 0.05) for subsequent NMF analysis. Based on the expression profile of these significant genes, we performed NMF analysis through the NMF package (20). Taking the front point of the maximum change of the cophenetic correlation with K as the best rank for NMF analysis, TCGA PRAD metabolism-related subgroups were obtained, and then dimensionality reduction visualization was carried out by using principal component analysis (PCA) and the t-distributed stochastic neighbor embedding (t-SNE) unsupervised clustering method was used to view the characteristics between different metabolic subgroups.

Moreover, we applied the expression profile of the same significant genes in ICGC to conduct NMF analysis using the same best rank as mentioned above. Then we used the SubMap module (21) of GenePattern (22) to map the subgroups obtained from TCGA and ICGC.

After obtaining the results of metabolism-related subgroups in TCGA and ICGC, we used the *survival* package (https://CRAN.R-project.org/package=survival to analyze the survival of different metabolic subgroups in the two datasets, as described previously (23), and compared the prognosis of the corresponding subgroups in the two different datasets.

### Identification of DEGs

In order to obtain the possible unique molecular biological functions of different metabolic subgroups, we analyzed the differences among three subgroups obtained from TCGA data. Differential analysis was performed based on the *limma* package (24), and the screening threshold of DEGs was |log_2_^Fold Change^| > 1 and adjusted P < 0.01.

Then, we used jveen, a flexible tool, to cross analyze the results of the three groups of DEGs (25) and obtain the unique DEGs of each subgroup relative to other subgroups, and then visualized the corresponding expression of these DEGs with a cluster heatmap.

### Functional Analysis

We used the Metascape database to enrich and analyze the unique DEGs of each subgroup (26) to explore the possible molecular biological functions, as described previously (27).

### GSVA

GSVA is a non-parametric and unsupervised gene set enrichment method that can estimate the score of a certain pathway or signature based on transcriptomic data (28). Similarly, we employed 2,752 metabolism-related genes from the previous study (19) to conduct GSVA using the gsva package (28). Then we got the score of each sample under 113 metabolic items and then conducted ANOVA among multiple subtypes based on the metabolic score. P < 0.01 was considered significant. For the significant metabolic items, we further conducted a Tukey posttest to judge the differences among different subgroups under the corresponding items. For each metabolic item, we selected the metabolic items with |diff| > 0.2 and adjusted P < 0.01 as the items with significant differences among different subgroups.

### Tumor Purity Analysis

In the tumor microenvironment, immune cells and stromal cells are two main types of tumor cells. ESTIMATE uses the expression profiles to predict the score of stromal cells and immune cells and then predicts the content of these two cells (29). Therefore, the tumor purity in each tumor sample can be calculated; that is, if there are many stromal cells and immune cells, the tumor purity is low, and on the contrary, the tumor purity is high. Here, we analyzed the tumor purity of TCGA based on the *estimate* package (https://R-Forge.R-project.org/projects/estimate, as described previously (30).

### Tumor Immune Microenvironment Analysis

CIBERSORT is a method to deconvolute the expression matrix of human immune cell subtypes based on the principle of linear support vector regression (31). It is mostly used for gene expression profiles, and the deconvolution analysis of unknown mixture and expression profiles containing similar cell types is better than other methods. Based on CIBERSORT, we obtained the composition of 22 immune cells in TCGA and statistically analyzed the immune microenvironment of different subtypes.

### Mutational Cancer Driver and Immune Gene Analysis

The Integrative OncoGenomics (IntOGen) pipeline is an implementation to obtain the compendium of mutational cancer drivers. Its application to somatic mutations of more than 28,000 tumors of 66 cancer types reveals 568 cancer genes and points toward their mechanism of tumorigenesis (32). We downloaded the cancer driver genes of PRAD from IntOGen and analyzed the main tumor driver gene (CGC_ CANCER_ Gene = TRUE) among subgroups. At the same time, we also analyzed the expression of major immune genes among subgroups.

### CNV, SNP, and Tumor-Specific Neoantigen Analysis

We downloaded the CNV data of TCGA PRAD from the GDAC Firehose database (http://gdac.broadinstitute.org/), as described previously (33), and then used the Gistic (version 2.0) module of GenePattern to analyze the CNV of each subgroup. The *maftools* package was used for visualization (34).

At the same time, we downloaded the PRAD somatic SNP results under the varscan processing flow from TCGA. After calculating the TMB, we used the *maftools* package (34) to statistically visualize the top 20 mutant genes and mutational cancer driver genes.

TSNAdb is a comprehensive tumor-specific neoantigen database based on pan-cancer immunogenomic analysis of somatic mutation data and human leukocyte antigen (HLA) allele information for 16 tumor types with 7,748 tumor samples from TCGA and The Cancer Immunome Atlas (TCIA) (35). We downloaded the tumor-specific neoantigens in PRAD and then analyzed the mutations carried by the tumor-specific neoantigens in the three subgroups.

## Results

### Patient Characteristics

Through acquisition and processing of raw data from TCGA and ICGC, we obtained 485 and 144 samples, respectively. The patient characteristics are listed in **Table 1**. After HGNC validation, we obtained 18,400 and 18,570 mRNAs in TCGA and ICGC, respectively (**Table S1**).

**Table 1:** Characteristics of patients in TCGA and ICGC datasets.

Variable	TCGA (N = 485)	ICGC (N = 144)
**Age (years)**		
≤60	219 (45%)	59 (41%)
>60	266 (55%)	85 (59%)
**Race**		
Asian	12 (2%)	Not applicable
Black or African American	56 (12%)	Not applicable
White	404 (83%)	Not applicable
Others	13 (3%)	Not applicable
**Gleason score**		
6	46 (9%)	29 (20%)
7	245 (51%)	80 (56%)
8	59 (12%)	9 (6%)
9	131 (27%)	2 (1%)
10	4 (1%)	Not applicable
Other	0 (0%)	24 (17%)
**Prostate-specific antigen (ng/ml)**		
<4	402 (83%)	Not applicable
≥4	26 (5%)	Not applicable
No detection	57 (12%)	Not applicable
**T stage**		
T1	0 (0%)	83 (58%)
T2	188 (39%)	61 (42%)
T3	280 (58%)	0 (0%)
T4	10 (2%)	0 (0%)
No detection	7 (1%)	0 (0%)
**N stage**		
N0	442 (91%)	Not applicable
N1	3 (1%)	Not applicable
No detection	40 (8%)	Not applicable
**M stage**		
M0	336 (69%)	Not applicable
M1	77 (16%)	Not applicable
No detection	72 (15%)	Not applicable
**Tumor status**		
Tumor free	338 (70%)	Not applicable
With tumor	88 (18%)	Not applicable
No detection	59 (12%)	Not applicable
**New event**		
No	357 (74%)	Not applicable
Yes	104 (21%)	Not applicable
No detection	24 (5%)	Not applicable
**Radiation therapy**		
No	383 (79%)	Not applicable
Yes	59 (12%)	Not applicable
No detection	43 (9%)	Not applicable
**Primary therapy outcome**		
Complete response	331 (68%)	Not applicable
Progressive disease	26 (5%)	Not applicable
Partial response	40 (8%)	Not applicable
Stable disease	29 (6%)	Not applicable
Other	59 (12%)	Not applicable
**Residual tumor**		
R0	206 (42%)	Not applicable
R1	144 (30%)	Not applicable
R2	5 (1%)	Not applicable
No detection	130 (27%)	Not applicable
**Zone of origin**		
Central zone	4 (1%)	Not applicable
Overlapping/multiple zones	124 (26%)	Not applicable
Peripheral zone	134 (28%)	Not applicable
Transition zone	7 (1%)	Not applicable
No detection	216 (45%)	Not applicable

Data were shown as n (%).

### Metabolism-Related PRAD Genes

We crossed 2,752 metabolism-related genes with 18,400 genes in TCGA PRAD to obtain 2,579 metabolism-related genes in PRAD. After further screening, 2,243 metabolism-related PRAD genes were left.

### NMF Analysis

**Figure 1:**
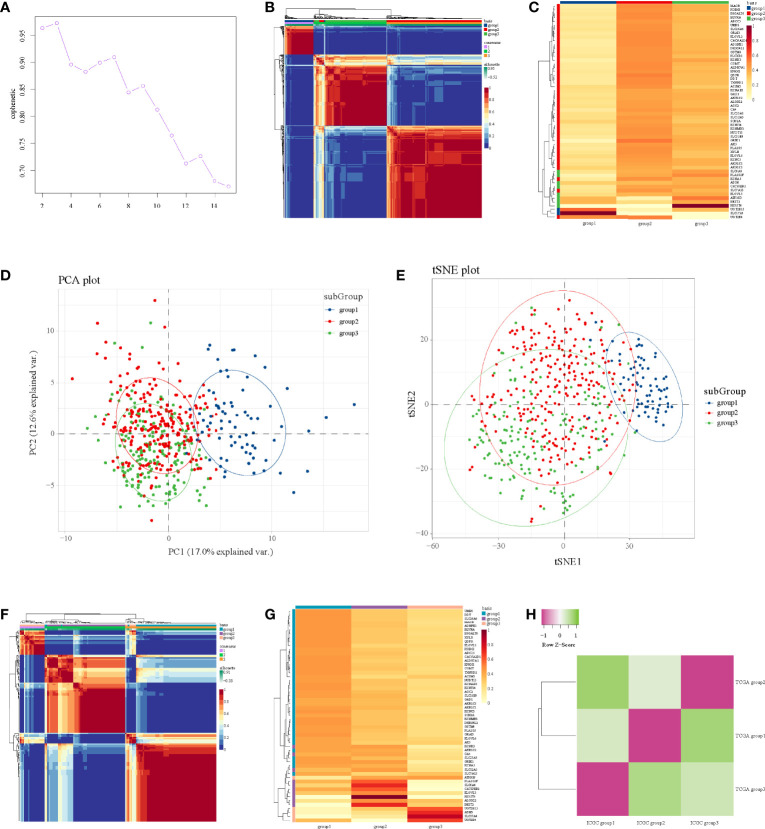
NMF analysis in the TCGA and ICGC datasets. **(A)** Identification of rank. **(B)** Heatmap of gene clustering of three subgroups in TCGA. **(C)** Heatmap of characteristic expression of three subgroups in TCGA. **(D)** Principal component analysis in TCGA. **(E)** T-distributed stochastic neighbor embedding in TCGA. **(F)** Heatmap of gene clustering of three subgroups in ICGC. **(G)** Heatmap of characteristic expression of three subgroups in ICGC. **(H)** Map of three subgroups in TCGA and ICGC assessed by the SubMap module of GenePattern.

We identified 56 significant genes for NMF after survival-related univariate regression analysis (**Table S2**). From **Figure 1A**, we identified three as the best rank for NMF analysis. Then, we divided 56 significant genes into three subgroups. A heatmap of the gene clustering method in TCGA showed significant differences in expression levels (**Figure 1B**). We also generated a heatmap to show the characteristic expression of 56 significant genes in the three subgroups (**Figure 1C**). The clinical relevance of the three subgroups is shown in **Figure S2**. The principal component analysis unsupervised clustering method indicated that the samples of different subgroups had significant characteristics, while subgroups 2 and subgroups 3 had certain similarities (**Figure 1D**). The same results were obtained by t-distributed stochastic neighbor embedding (t-SNE) (**Figure 1E**).

Then, we used the data from ICGC to verify these three subgroups in TCGA; we also used three as the rank for NMF analysis of the expression profile of 56 significant genes in ICGC to obtain three subgroups, and the internal consistency of the three subgroups was also good (**Figure 1F**). **Figure 1G** shows the characteristic expression of 56 significant genes in the three subgroups in ICGC. Through mapping the three subgroups from TCGA and ICGC, we confirmed their corresponding relationships (**Figure 1H**). Detailly, TCGA subgroup 3 corresponded to ICGC subgroup 2, TCGA subgroup 1 corresponded to ICGC subgroup 3, and TCGA subgroup 2 corresponded to ICGC subgroup 1 (**Figure 1H**). Importantly, the corresponding relationships were consistent with the characteristic expression of 56 significant genes in the subgroups (**Figures 1C, G**), indicating that our submap matching analysis results of subgroups in the two datasets were good.

### Survival Analysis

**Figure 2:**
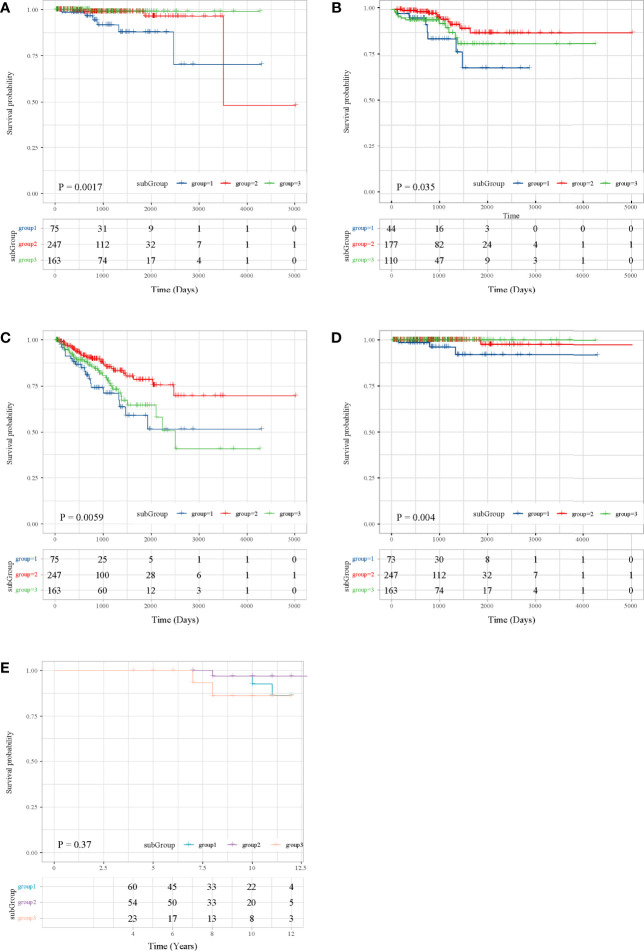
Survival analysis in the TCGA and ICGC datasets. **(A)** OS in TCGA. **(B)** DFI in TCGA. **(C)** PFI in TCGA. **(D)** DFS in TCGA. **(E)** OS in ICGC.

Then, we performed survival analysis under subgroups in both TCGA and ICGC to explore the prognosis. According to TCGA data, the three subgroups had statistically significant overall survival differences (*P* = 0.0017, **Figure 2A**), and we can observe that the overall survival of subgroup 1 was the worst, subgroup 2 was the second, subgroup 3 has a better overall survival, and the overall survival of subgroup 2 and subgroup 3 was the same. Moreover, patients in subgroup 1 shared an unfavorable disease-free interval (*P* = 0.035, **Figure 2B**), progression-free interval (*P* = 0.0059, **Figure 2C**), and disease-specific survival (*P* = 0.004, **Figure 2D**). In ICGC, the overall survival of subgroup 3 was the worst, subgroup 1 was the second, subgroup 2 was the best, and the overall survival of subgroup 1 and subgroup 2 was the same (**Figure 2E**). These results were consistent with the results of subgroup matching of the two datasets (**Figure 1H**).

### Functional Analysis of DEGs

**Figure 3:**
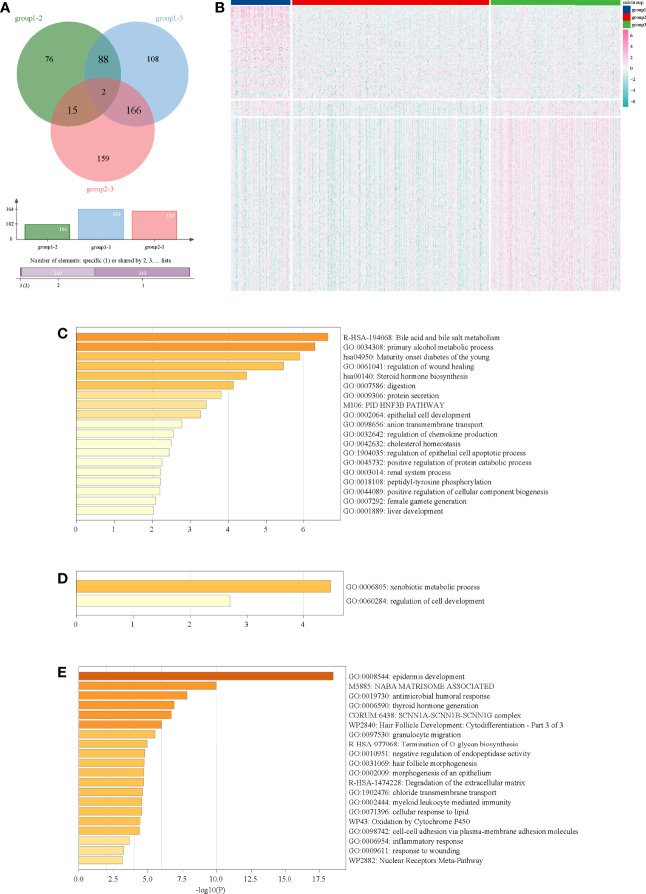
Functional analysis of DEGs in three subgroups. **(A)** Venn diagram of DEGs in three subgroups. **(B)** Heatmap of DEGs in three subgroups. **(C)** Functional analysis of DEGs in subgroup 1. **(D)** Functional analysis of DEGs in subgroup 2. **(E)** Functional analysis of DEGs in subgroup 3.

Through multi-subgroup DEG screening, we obtained 88 DEGs in subgroup 1, 15 DEGs in subgroup 2, and 166 DEGs in subgroup 3 (**Figure 3A**). Moreover, we found that most of the DEGs in subgroup 1 and subgroup 3 were significantly up-regulated, whereas most of the DEGs in subgroup 2 were down-regulated (**Figure 3B**). Later functional analysis indicated that these DEGs in the three subgroups were enriched with some metabolism-related biological processes and pathways (**Figures 3C–E**).

### GSVA

**Figure 4:**
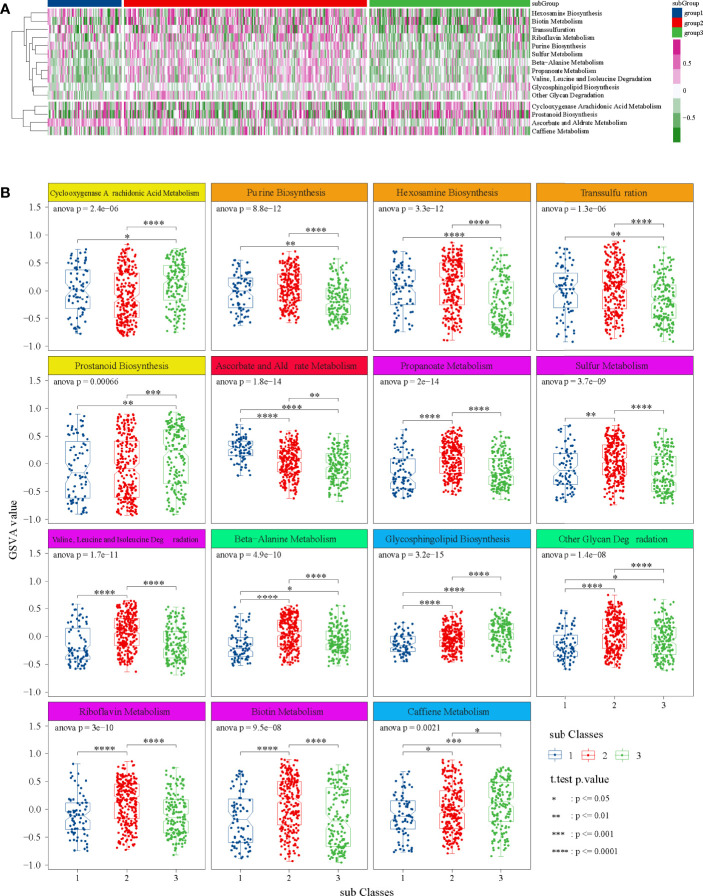
GSVA in TCGA dataset. **(A)** Heatmap of 15 significant metabolic items in three subgroups. **(B)** Box diagrams of 15 significant metabolic items in three subgroups. ANOVA test was performed among groups, and t-test was performed between the two groups.

Through preliminary screening, we obtained 15 significant metabolic items (**Figure 4A**). Among them, for most metabolic pathways, the metabolism of subgroup 2 was higher than that of subgroup 1 and subgroup 3. Detailly, cyclooxygenase arachidonic acid metabolism and ascorbate and aldrate metabolism were the highest in subgroup 1, whereas prostanoid biosynthesis; propanote metabolism; valine, leucine, and isoleucine degradation; beta-alanine metabolism; glycosphingolipid biosynthesis; other glycan degradation; and caffeine metabolism were the lowest in subgroup 1 (**Figure 4B**).

### Tumor Purity and Immune Microenvironment Analysis

**Figure 5:**
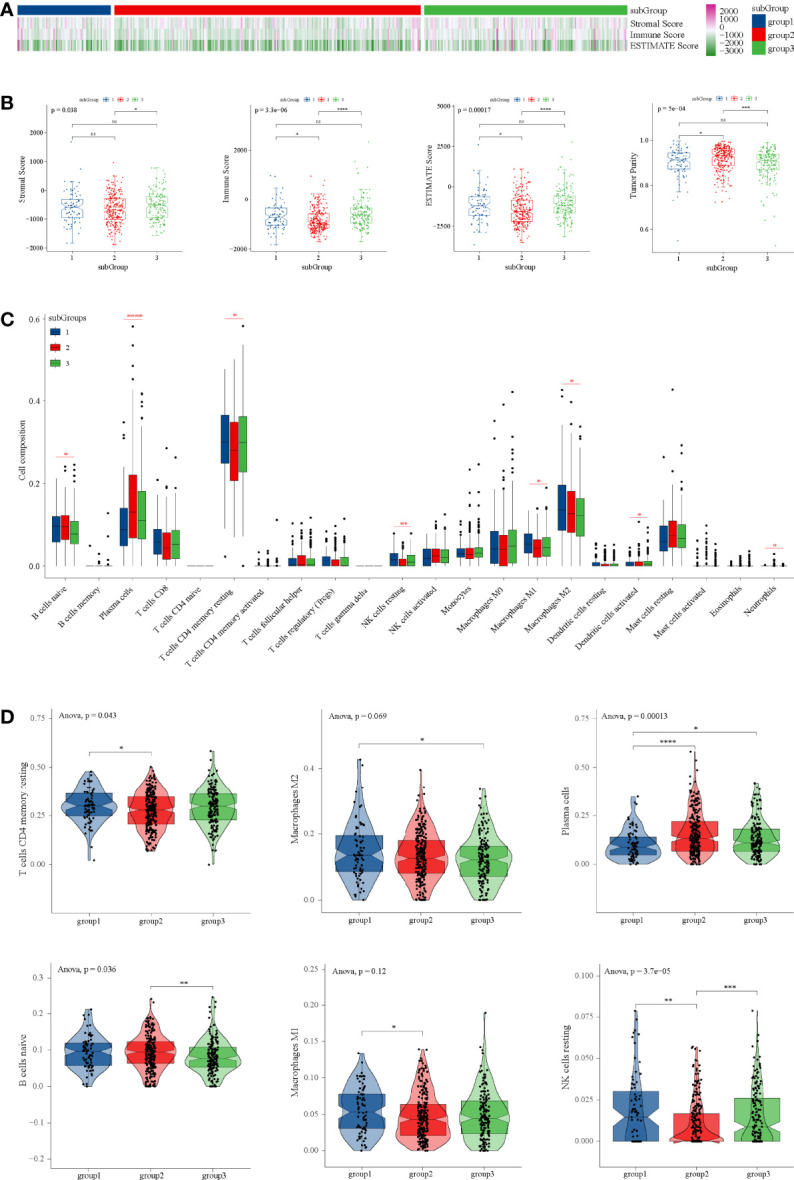
Tumor purity and immune microenvironment analysis in TCGA dataset. **(A)** Heatmap of tumor purity analysis in three subgroups. **(B)** Box diagrams tumor purity analysis in three subgroups. **(C)** Composition of 22 immune cells in TCGA. **(D)** Immune cell types with significant differences among subgroups. ANOVA test was performed among groups, and t-test was performed between the two groups. *P < 0.05, **P < 0.01, ***P < 0.001, ****P < 0.0001 and ns P ≥ 0.05.

The results indicated that the tumor purity of subgroup 2 was significantly higher than that of subgroup 1 and subgroup 3, and the composition of immune cells of subgroup 2 was lower than that of subgroup 1 and subgroup 3 (**Figures 5A, B**).

Through CIBERSORT, we found that the main immune cell type is T cells CD4 memory resetting, followed by macrophage M2, plasma cells, and B cells naïve in TCGA (**Figure 5C**). T cells CD4 memory resting, which were the main components of PRAD, were significantly lower than subgroup 1 and subgroup 3 (**Figure 5D**), which was consistent with tumor purity. However, in subgroup 2, there were significantly more plasma cells than in subgroup 1 and subgroup 3 (**Figure 5D**).

### Mutational Cancer Driver and Immune Gene Analysis

From IntOGen, we obtained 11 mutational cancer driver genes in PRAD. Among these genes, CDKN2A was down-regulated in subgroup 1, BRCA2 was down-regulated in subgroup 2, and BNF43 was up-regulated in subgroup 2 (**Figure S3A**). Moreover, we demonstrated that the expression of major immune genes, such as CCL2, CD274, CD276, CD4, CTLA4, CXCR4, IL1A, IL6, LAG3, TGFB1, TNFRSF4, TNFRSF9, and PDCD1LG2, in subgroup 2 was almost significantly lower than that in subgroup 1 and subgroup 3 (**Figure S3B**), which is consistent with the results of tumor purity analysis.

### CNV, SNP, and Tumor-Specific Neoantigen Analysis

**Figure 6:**
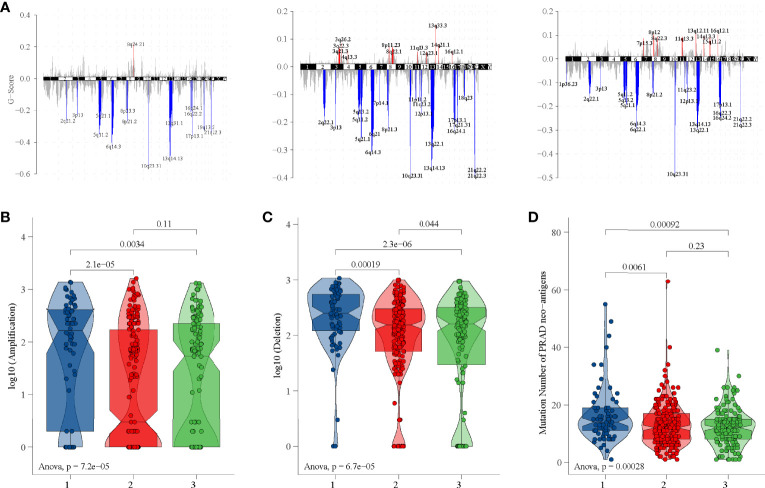
CNV and tumor-specific neoantigens analysis in TCGA dataset. **(A)** G-scores of three subgroups. **(B)** Copy number amplification of three subgroups. **(C)** Copy number deletion of three subgroups. **(D)** Tumor-specific neoantigens of three subgroups.

Each area of CNV is assigned a G-score that considers the amplitude of the alteration as well as the frequency of its occurrence across samples (36). Subgroup 1 had amplifications of 8q24.21, subgroup 2 had amplifications of 13q33.3, 3q26.2, and 8q22.1, and subgroup 3 had amplifications of 13q12.11, 8p12, and 8q22.3 (**Figure 6A** and **Table S3**). Interestingly, all subgroups had deletions of 10q23.31, 3p13, 5q11.2, 5q21.1, 6q14.3, 13q14.13, 17p13.1, and 21q22.3 (**Figure 6A** and **Table S3**). In addition, subgroup 1 had the highest copy number amplification (**Figure 6B**) and copy number deletion (**Figure 6C**). Moreover, the tumor-specific neoantigens of subgroup 1 carried more mutations than subgroup 2 and subgroup 3 (**Figure 6D**).

**Figure 7:**
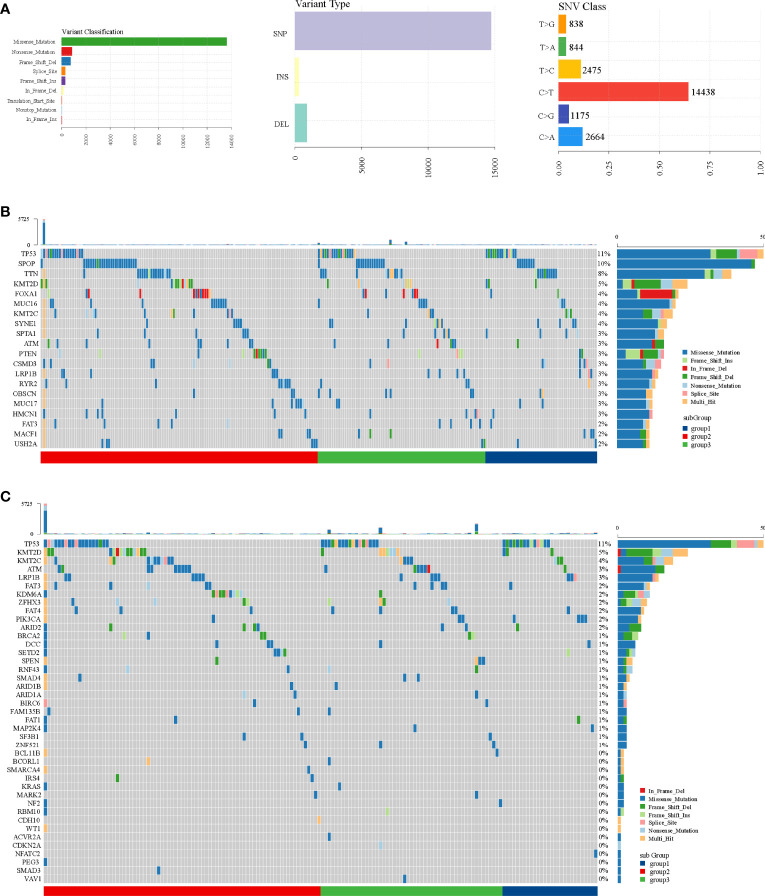
SNP analysis in TCGA dataset. **(A)** Variant classification, variant type, and SNV class. **(B)** Oncoplot of top 20 mutant genes. **(C)** Oncoplot of mutational cancer driver genes.

Through SNP analysis, missense mutation was the most frequent variant classification and C>T was the most frequent single-nucleotide variant (**Figure 7A**). From the oncoplots of top 20 mutant genes (**Figure 7B**) and mutational cancer driver genes (**Figure 7C**), we found that TP53, FAT3, LRP1B, ATM, KMT2C, and KMT2D from mutant genes were also recognized as cancer driver genes.

## Discussion

In recent years, the heterogeneity of PCa has been an important topic of research (37). Exploring new tumor subtypes, especially combined metabolism, is an effective way to study their heterogeneity and thus provides insights for clinicians to conduct more accurate clinical evaluation. Bioinformatics analysis based on database has increasingly shown its superiority and clinical applicability (23, 38). Here, we used metabolism-related genes to conduct consensus clustering among large-scale PCa patients and finally identified three PCa subgroups. These subgroups were validated by an external clinical patient cohort from ICGC. We conducted GSVA to enrich pathways. ESTIMATE and CIBERSORT algorithms were used to conduct an integrative analysis of immune scores and immune cells in the different subgroups. Finally, cancer driver genes, CNV, SNP, and tumor-specific neoantigens were analyzed. Our results from this study might reveal the molecular mechanism of metabolism-induced PCa.

Through survival analysis, we found that the prognosis of patients in subgroup 1 was significantly worse than that of the other two subgroups in TCGA, and there was no significant difference in prognosis among other two subgroups. Moreover, the prognosis of patients in subgroup 3 (corresponded to TCGA subgroup 1) was significantly worse than that of the other two subgroups in ICGC and there was no significant difference in prognosis among other two subgroups. Principal component analysis (PCA) and t-distributed stochastic neighbor embedding (t-SNE) showed a clear boundary between these subgroups in both TCGA and ICGC.

Steroidogenic enzymes are essential for PCa development (39). 17βHSD2 decreased potent androgen production by converting testosterone or dihydrotestosterone to each of their upstream precursors (40), which might provide new strategies for clinical management. On the other hand, chemokine signaling regulates tumor metastasis (41). CXCL12, a member of the chemokine family, and its receptor, CXCR4, are key mediators of PCa bone metastasis (42). What is more, the tyrosine 190 and 211 phosphorylation of proliferation cell nuclear antigen is a frequent event in advanced prostate cancer (43, 44). Strikingly, we found that steroid hormone biosynthesis, regulation of chemokine production, and peptidyl-tyrosine phosphorylation were enriched in subgroup 1 in TCGA. Cyclooxygenase is a rate-limiting enzyme involved in the cyclooxygenase metabolic pathway of arachidonic acid, which can catalyze the conversion of arachidonic acid to prostaglandins (45). A systematic review indicated that an 8.75-month increase in progression-free survival and an improved trend in overall survival in the cancers received ascorbate (46). Interestingly, our study identified that cyclooxygenase arachidonic acid metabolism and ascorbate and aldrate metabolism were the highest in subgroup 1. Altogether, our results explain the poor prognosis of this particular population.

Patients in subgroup 1 had the highest tumor purity, causing worse prognosis. We also found that T cells CD4 memory resetting, macrophages M2, macrophages M1, B cells naïve, and NK cells resetting infiltrated significantly more in subgroup 1. Previous data showed that PRAD patients with high numbers of M2 macrophages in the tumor environment had increased odds of dying (47). These cells could promote PCa progression by promoting immunosuppressive responses (48, 49). Through CNV analysis, we found that 8q24.21, strongly associated with risk of tumors (50, 51), was amplificated in subgroup 1. Some long non-coding RNAs, such as CCAT1 (52, 53), PVT1 (54), PRNCR1 (55), and PCAT1 (56), in 8q24.21 have an influence in oncogenesis of PCa. The tumor-specific neoantigens might contribute to the immunogenic phenotype (57). In prostate cancer, inactivating CDK12 mutations produces tumor-specific neoantigens and possibly sensitivity to immunotherapy (58). Vaccines that target tumor-specific neoantigens have the potential to induce robust antitumor responses (59). Sipuleucel-T, a neoantigen vaccine, prolonged overall survival among men with metastatic castration-resistant prostate cancer (60). In the present study, we confirmed more mutations of tumor-specific neoantigens in subgroup 1, which may be one of the reasons for the poor prognosis.

There are some limitations in this study. First, the available public datasets in our study were from TCGA and ICGC, two independent data platforms. Although the results can be well verified, there is inevitable selection bias. We will further validate our results in the inpatients at our hospitals. Second, limited by the survival data in ICGC, we could not conduct multiple survival analyses like TCGA; we can only do OS analysis. Moreover, although we used different datasets as the training cohort and validation cohort respectively, the sample size of each cohort is still small, and large-sample data are needed to verify our findings. Finally, more solid experimental studies are needed to verify DEGs and their immune and molecular biological mechanism.

## Conclusion

Collectively, the metabolic mechanism of PCa was systematically explored, providing associations with mutational burden and immune infiltrations. PCa-related signatures prove to have advantages in predicting prognosis and can be used as a good molecular classifier to find different metabolic types. The relevant findings will need further basic experiments and even clinical trials to be corroborated in the future before they can be further applied in clinical practice.

## Data Availability Statement

The original contributions presented in the study are included in the article/**Supplementary Material**. Further inquiries can be directed to the corresponding authors.

## Author Contributions

GY, BL, and KY contributed equally as first authors. BX, TJ, QY, and YL contributed equally as senior authors. GY, BX, and MZ conceived, designed, or planned the idea. XG, JW, and SS contributed to the language editing of the manuscript. GY, BL, and KY analyzed the data. All authors collected the data. All authors interpreted the results. GY and BL wrote the manuscript. BX, TJ, QY, and YL revised the manuscript. All authors contributed to the article and approved the submitted version.

## Funding

The study is supported by the National Natural Science Foundation of China (82072846) to BX, the Innovative Research Team of High-Level Local Universities in Shanghai (19DZ2204000) to BX, the Natural Science Foundation of Shanghai (22ZR1437300) to GY, the Interdisciplinary Program of Shanghai Jiao Tong University (project number: YG2019QNA12) to GY, the Shanghai Anticancer Association EYAS PROJECT (SACA-CY19C03) to GY, and the Fundamental Research Program Funding of Ninth People’s Hospital affiliated to Shanghai Jiao Tong University School of Medicine(JYZZ006) to GY.

## Conflict of Interest

The authors declare that the research was conducted in the absence of any commercial or financial relationships that could be construed as a potential conflict of interest.

Reviewer DH declared a shared parent affiliation with authors GY, MZ, XG, JW, SS, YL, QY, TJ, and BX to the handling editor at the time of review.

## Publisher’s Note

All claims expressed in this article are solely those of the authors and do not necessarily represent those of their affiliated organizations, or those of the publisher, the editors and the reviewers. Any product that may be evaluated in this article, or claim that may be made by its manufacturer, is not guaranteed or endorsed by the publisher.
