# Supplementary material for: Identification of Metabolism-Related Gene-Based Subgroup in Prostate Cancer
Source: Front Oncol. 2022 Jun 16;12:909066. doi: 10.3389/fonc.2022.909066 (PMC9243363; doi:10.3389/fonc.2022.909066)
Supplement: Supplementary file 1 [file DataSheet_1.docx]

Supplementary Material

# Supplementary Figures


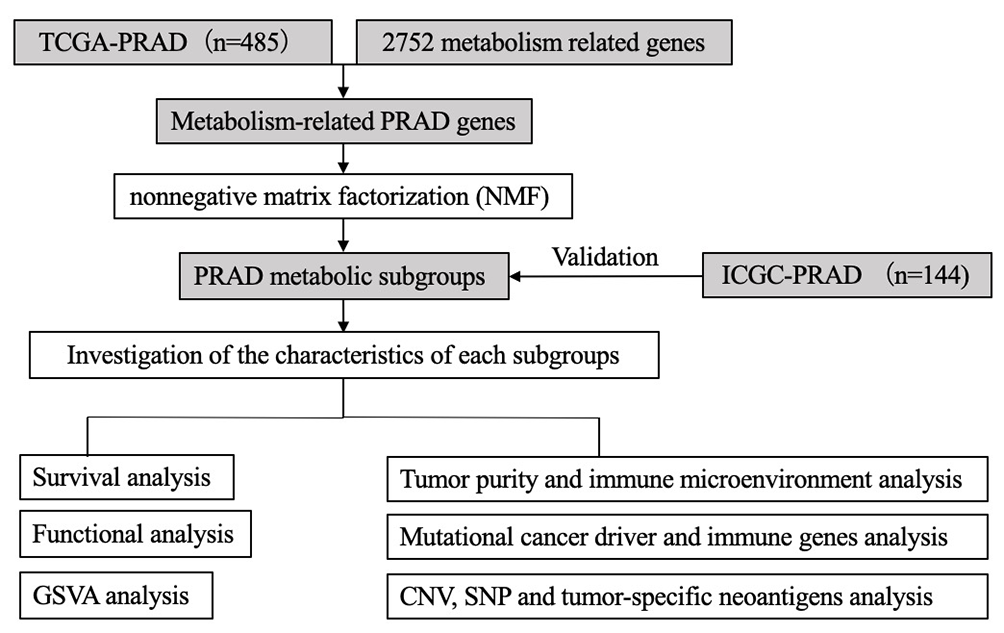


**Figure S1. Work flow of the study.**


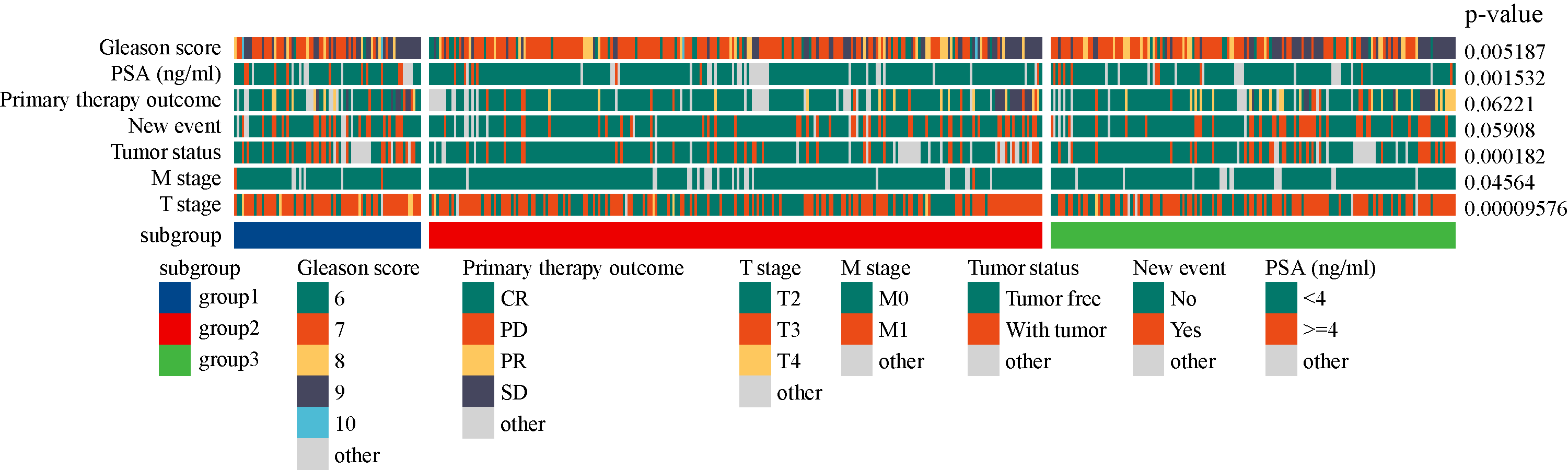


**Figure S2. Clinical correlation analysis in TCGA.**


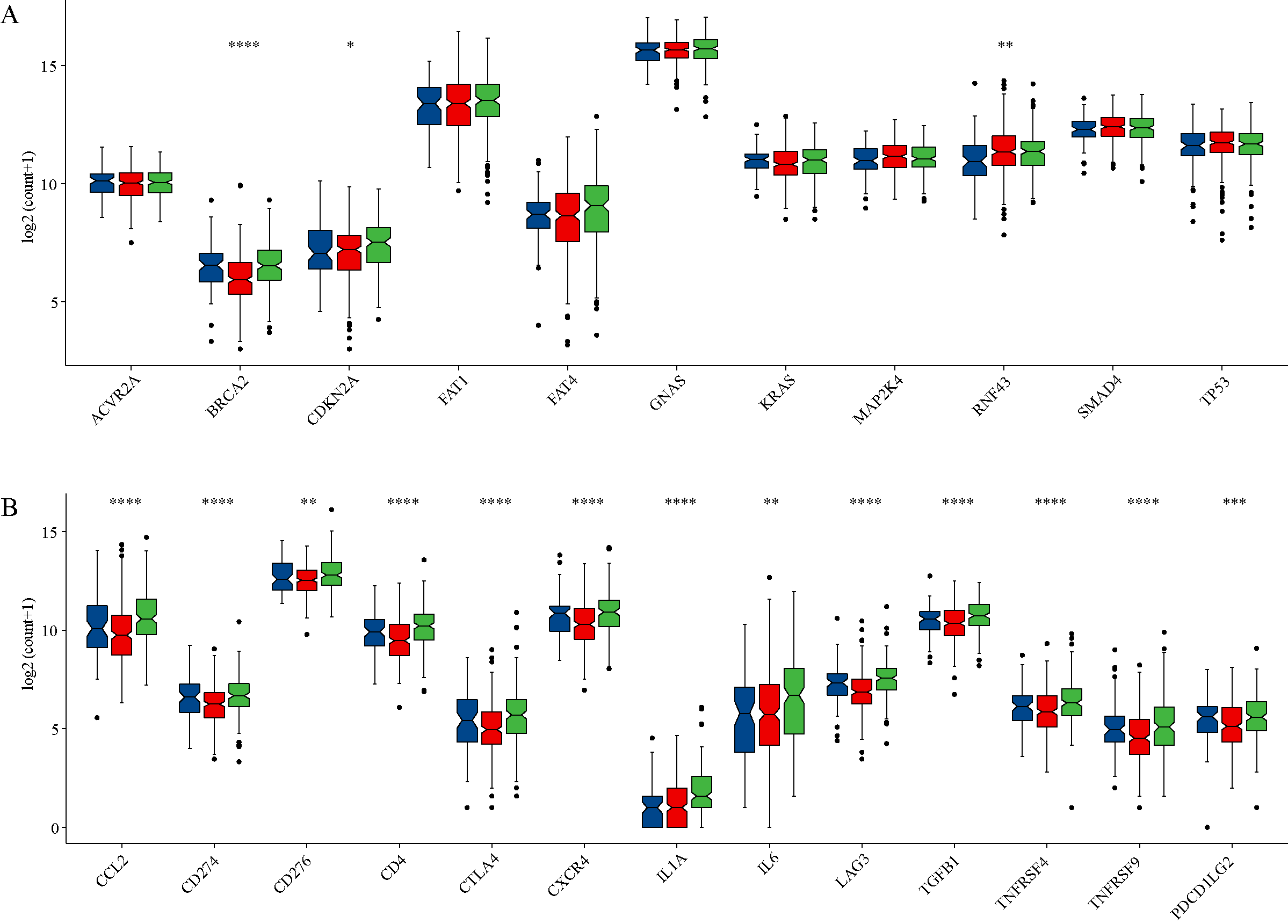


**Figure S3. Mutational cancer driver (A) and immune genes (B) analysis in TCGA.**

# Supplementary Tables

**
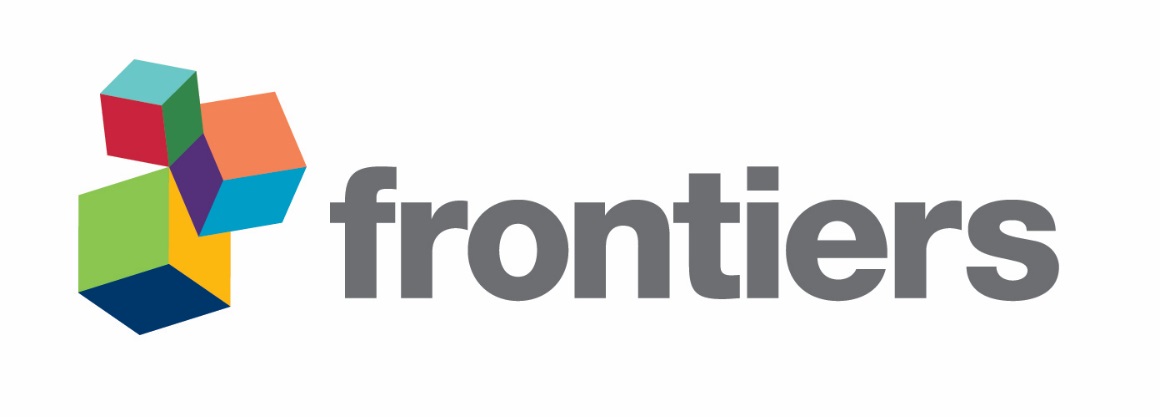
**

**Table S1. Details of genes in TCGA and ICGC.**

**Table S2. Details of 56 genes used for NMF.**

**Table S3. CNV analysis of three subgroups.**
